# New regulators of the tetracycline‐inducible gene expression system identified by chemical and genetic screens

**DOI:** 10.1002/2211-5463.13482

**Published:** 2022-09-11

**Authors:** Valeria Colicchia, Maria Häggblad, Oleksandra Sirozh, Bartlomiej Porebski, Mirela Balan, Xuexin Li, Louise Lidemalm, Jordi Carreras‐Puigvert, Daniela Hühn, Oscar Fernandez‐Capetillo

**Affiliations:** ^1^ Science for Life Laboratory, Division of Genome Biology, Department of Medical Biochemistry and Biophysics Karolinska Institute Stockholm Sweden; ^2^ Genomic Instability Group Spanish National Cancer Research Centre (CNIO) Madrid Spain; ^3^ Present address: Department of Biology University of Rome Tor Vergata Italy; ^4^ Present address: Department of Pharmaceutical Biosciences and Science for Life Laboratory Uppsala University Sweden

**Keywords:** ALS, chemical screen, doxycycline, TDP‐43, tetR

## Abstract

The tetracycline repressor (tetR)‐regulated system is a widely used tool to specifically control gene expression in mammalian cells. Based on this system, we generated a human osteosarcoma cell line, which allows for the inducible expression of an EGFP fusion of the TAR DNA‐binding protein 43 (TDP‐43), which has been linked to neurodegenerative diseases. Consistent with previous findings, TDP‐43 overexpression led to the accumulation of aggregates and limited the viability of U2OS. Using this inducible system, we conducted a chemical screen with a library that included FDA‐approved drugs. While the primary screen identified several compounds that prevented TDP‐43 toxicity, further experiments revealed that these chemicals abrogated the doxycycline‐dependent TDP‐43 expression. This antagonistic effect was observed with both doxycycline and tetracycline, and in several Tet‐On cell lines expressing different genes, confirming the general effect of these compounds as inhibitors of the tetR system. Using the same cell line, a genome‐wide CRISPR/Cas9 screen identified epigenetic regulators such as the G9a methyltransferase and TRIM28 as potential modifiers of TDP‐43 toxicity. Yet again, further experiments revealed that G9a inhibition or TRIM28 loss prevented doxycycline‐dependent expression of TDP‐43. In summary, we have identified new chemical and genetic regulators of the tetR system, thereby raising awareness of the limitations of this approach to conduct chemical or genetic screening in mammalian cells.

## Graphical abstract

Promoters harbouring tetO sequences enable the regulated expression of a gene of interest in response to tetracycline and related chemicals. By conducting chemical and genetic screens, Colicchia *et al*. identify novel drugs and mutations that impair tet‐dependent gene expression. These findings raise awareness of the potential limitations of using Tet‐regulated gene expression systems for conducting phenotypic screens.

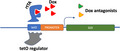

**Abbreviations**
ALS: amyotrophic lateral sclerosis
BFP: blue fluorescent protein
BrM: bromocriptine mesylate
cDNA: complementary DNA
CRISPR: clustered regularly interspaced short palindromic repeats
DNA: deoxyribonucleic acid
Dox: doxycycline
EGFP: enhanced green fluorescent protein
FDA: U.S. Food and Drug Administration
GOI: gene of interest
HTM: High‐throughout microscopy
Lop: loperamide
MoF: mometasone furoate
MOI: multiplicity of infection
mRNA: messenger RNA
NGS: next‐generation sequencing
Nig: niguldipine
qRT–PCR: quantitative reverse transcription–polymerase chain reaction
RNA: ribonucleic acid
SEM: standard error of the mean
sgRNA: single guide RNA
TDP‐43: TAR DNA‐binding protein 43
Tet: tetracycline
tetO: Tet operator
tetR: tetracycline repressor
UMI: unique molecular identifier

Amyotrophic lateral sclerosis (ALS) is a progressive and fatal neurodegenerative disease that selectively affects motor neurons and currently lacks a cure [1]. Even if ALS has been associated with a very broad range of mutations, a common hallmark is the accumulation of abnormal ubiquitinated aggregates frequently containing TDP‐43 [2]. The discovery of mutations in the gene coding for TDP‐43 (*TARDBP*), further supported a role for TDP‐43 dysfunction in ALS [3, 4]. TDP‐43 has a strong affinity for RNA and has been involved in many reactions involving RNA such as translation, splicing or transport [5]. Despite intense research in this area, how TDP‐43 dysregulation is involved in neurodegeneration is still not completely understood. Intriguingly, both loss or overexpression of TDP‐43 is toxic, and this property has been used to generate experimental models of ALS [6, 7, 8]. Such models have led to the discovery of mutations in proteins such as ATXN‐2 [9] or in components of the autophagosome‐lysosome pathway [10] as modifiers of TDP‐43 toxicity. By contrast, no chemical therapy has been yet identified that significantly rescues TDP‐43 toxicity.

The tetracycline repressor (tetR)‐regulated system is extensively used to regulate the inducible expression of specific genes of interest (GOI) in eukaryotic cells by the addition or withdrawal of tetracycline antibiotics [11]. These systems are based on the binding of the tetR protein to the tet operator (tetO), first discovered in the tetracycline resistance operon encoded by the Tn10 transposon of *Escherichia coli* [12]. While tetR bound to tetracycline is a transcriptional repressor, in the so‐called Tet‐ON system a tetR variant containing four mutations allows for the inducible expression of the GOI in response to tetracycline antibiotics [13]. Since its first application in eukaryotic cells [14], the tetR‐regulated system was further optimized until it became a standard method in the toolbox of molecular biologists, being extensively used for inducible gene expression in both *in vitro* and *in vivo* experiments. Noteworthy, and despite its usefulness, the use of tetracycline antibiotics for inducing the expression of a GOI might also have unintended effects such as promoting alterations of cell metabolism and gut microbiota, delaying plant growth and the inhibition of cell proliferation and mitochondrial protein translation [15, 16, 17, 18, 19, 20]. Here, we report the development of a Tet‐ON cell model, which allows for inducible TDP‐43 expression. Using this system, we conducted chemical and CRISPR‐Cas9‐based genome‐wide forward genetic screens aiming to identify new modulators of TDP‐43 toxicity.

## Materials and methods

### Plasmids

EGFP and TDP‐43^EGFP^ cDNAs were amplified from pEGFP‐C1 (Takara Bio, San Jose, CA, USA, 6084–1) and pEGFP‐C1‐TDP‐43 (kind gift from Dr. Tatiana Shelkovnikova, Cardiff University, UK) vectors, respectively. Each amplified cDNA was cloned into pINTO‐C‐HF (kind gift from Dr. Emilio Lecona, Centro de Biología Molecular Severo Ochoa, Spain) to obtain pINTO‐EGFP and pINTO‐wtTDP‐43^EGFP^ plasmids, respectively. The (PR)_97_ construct was a kind gift from Oleksandra Sirozh (CNIO). The sgATXN2 (AATCTATGCAAATATGAGGA) was cloned into pX458 [21], according to the strategy outlined in the above paper, and cloning was confirmed by sequencing. The cDNA of ATXN2 was amplified from pcDNA‐HA‐ATXN2 (gift from Prof. Daisuke Ito, Keio University, Japan), and cloned into Rosa‐BleoR‐TetON‐Snap, which was obtained by removing the OsTIR1cDNA construct from the pROSA26‐DV1_OsTIR vector (kind gift from Dr. Bennie Lemmens, Karolinska Institutet, Sweden).

### Cell lines

T‐REx U2OS cells were grown in DMEM Glutamax medium (Gibco™, Thermo Fisher Scientific, Waltham, MA, USA, 31966021) supplemented with 10% tetracycline‐free FBS (Takara Bio USA, Inc., San Jose, CA, 631106), 1% Penicillin/Streptomycin and 2 μg·mL^−1^ blasticidin. T‐REx U2OS cells were transfected with plasmids carrying EGFP, TDP‐43^EGFP^ and (PR)_97_ using Lipofectamine3000 (Thermo Fisher Scientific) according to the manufacturer's instructions. After 72 h, transfected cells were selected in zeocin (0.2 mg·mL^−1^) for 10 days. Individual resistant clones were tested for transgene expression by WB and IF with 1 μg·mL^−1^ of Dox. hTERT RPE‐1 cells were first transfected with the vector pX458 carrying the sgRNA targeting ATXN2. 72 h after transfection, EGFP‐positive cells were sorted and plated at low density for the selection of resistant clones. ATXN2‐deficient clones were validated by IF and WB. A selected clone was next co‐transfected with Rosa‐BleoR‐TetON‐SnapATXN2wt and sgRosa‐pX458 for targeted insertion into the Rosa locus. EGFP‐positive cells were sorted and selected with 0.5 mg·mL^−1^ zeocin. Selected clones were stained with ATXN2 antibody and those with high and homogenous expression were expanded and frozen. For the generation of TRIM28 deficient clones, U2OS^T43^ cells were co‐transfected with Lipofectamine 2000 with the following constructs a crRNA targeting exon 5 of TRIM28 (Horizon Discovery, Cambridge, UK, CM‐005046‐01‐0002), the transactivating CRISPR RNA, tracrRNA: (Horizon, U‐002005‐05) and pSpCas9(BB)‐2A‐GFP (PX458) (Addgene, Watertown, MA, USA, #48138), following the Edit‐R™ CRISPR‐Cas9 Gene Engineering System (Dharmacon, Horizon Discovery). 48 h after transfection, Cas9_GFP positive cells were sorted and seeded at the low confluence. After 1 week, clones lacking TRIM28 expression were identified by WB and IF. SH‐SY5Y cells (ATCC) were maintained in DMEM/F12 supplemented with 10% FCS and penicillin/streptomycin. For neuronal differentiation, SH‐SY5Y cells were seeded on laminin‐coated 96‐well imaging plates in DMEM/F12 with 5% FCS. Next day, the medium was changed to DMEM/F12 containing 1% FCS and 10 mm retinoic acid. After 5 days, cells were treated with indicated compounds for 48 h.

### High‐throughput chemical screen

U2OS^T43^ cells were seeded at 1000 cells per well, with or without Dox (10 ng·mL^−1^), in 384‐well plates (BD Falcon, Franklin Lakes, NJ, USA, 353962) and incubated at 37 °C in 5% CO_2_ for 24 h. On the next day, compounds were added to triplicate plates to get a final concentration of 1 μm and incubated for further 48 h. Cells were then fixed and stained with 4% formaldehyde and 2 μm Hoechst for 20 min. Plates were imaged using an InCell Analyzer 2200 high‐content microscope with a 4× objective covering the entire field of each well. Nuclei were counted using CellProfiler (www.cellprofiler.org). Analysis of high‐content imaging data was performed using KNIME Analytics (www.knime.com) and additional statistical analysis was carried out using Microsoft Excel and graphpad prism (San Diego, CA, USA) softwares. Plate and liquid handling were performed using Echo®550 (Labcyte, San Jose, CA, USA), MultiFlo Dispenser (Integra, Mettler Toledo, Columbus, AL, USA), VIAFLO 384 (Integra), HydroSpeed plate washer (Tecan, Männedorf, Switzerland) and Paa Microplate Handler KiNEDEx BX‐470 robot. The chemical collection was provided by the Chemical Biology Consortium Sweden (CBCS) and consisted of 4221 pharmacologically active compounds from the following libraries: Enzo, Prestwick, Selleck tool compounds, Selleck‐known kinase inhibitors, SGC Bromodomains and Tocris.

For the secondary screen, U2OS^T43^ cells were again seeded at 1000 cells per well, with or without Dox (10 ng·mL^−1^), in 384‐well plates (BD Falcon, 353962) and incubated at 37 °C in 5% CO_2_ for 24 h. On the following day, the 17 compounds identified as hits in the primary screen were added at three different concentrations (1, 5 and 10 μm) and incubated for 48 h. Cells were fixed and stained with 4% formaldehyde and 2 μm Hoechst for 20 min. Fixed cells were permeabilized with 0.2% Triton X‐100 in PBS for 10 min and then blocked by 3%BSA + 0.1% Tween20 in PBS for 30 min. An anti‐TDP‐43 antibody (Abcam, Cambridge, UK, ab41881) was diluted 1 : 250 in blocking buffer and incubated at +4 °C under gentle shaking overnight. The anti‐rabbit Alexa Fluor 647 secondary antibody (Invitrogen, Thermo Fisher Scientific, A32733) was diluted 1 : 1000 in blocking buffer and incubated for 40 min at room temperature. After washing, plates were imaged using the InCell Analyzer 2200 with a 20× objective. Nuclei counts, and the levels of TDP‐43^EGFP^ and endogenous TDP‐43 were quantified using CellProfiler and data analysed using the KNIME Analytics Platform.

### Chemicals

Doxycycline hyclate (Dox, D9891), tetracycline hydrochloride (tet, T7660), mometasone furoate (M4074) and BIX01294 trihydrochloride hydrate (B9311) were purchased from Sigma Aldrich (St. Louis, MO, USA). Loperamide hydrochloride (ALX‐550‐253) and niguldipine hydrochloride (BML‐CA216) were purchased from Enzo Life Sciences (New York, NY, USA). Bromocriptine mesylate (0427) was purchased from Tocris (Bristol, UK). Zeocin (R25001) and blasticidin (R21001) were purchased from Thermo Fisher Scientific.

### qRT–PCR

Total RNA was isolated using PureLink RNA mini kit (Invitrogen) according to the manufacturer's protocols, and RNA was quantified by NanoDrop Lite Spectrophotometer (Thermo Scientific). 50 ng of RNA for each sample was retrotranscribed and amplified by Power SYBR® Green RNA‐to‐CT™ 1‐Step Kit (Thermo Scientific, 4389986), using specific primers for EGFP. GAPDH was used as housekeeping gene control. Primer sequences, together with the source of the rest of the reagents used in this study are provided in Table S3.

### Immunofluorescence

U2OS^PR97^ and RPE‐1^ATXN2^ cells were fixed and permeabilized with 0.2% Triton X‐100 diluted in PBS for 10 min, blocked in 3%BSA containing 0.1% Tween20 in PBS for 30 min and incubated overnight with antibodies against PR repeats (Proteintech, Manchester, UK, 23979‐1‐AP; 1 : 500) and ATXN2 (BD Biosciences, Franklin Lakes, NJ, USA, 611378; 1 : 1000). Anti‐rabbit and anti‐mouse Alexa Fluor 647 secondary antibodies (Invitrogen, A32733 and A32728) were diluted 1 : 1000 in blocking buffer and incubated 40 min at room temperature. After washing, plates were imaged using the InCell Analyzer 2200 microscope with a 20× objective. Integrated intensities were evaluated using CellProfiler pipeline, and data were analysed using the KNIME Analytics Platform.

For the evaluation of TDP‐43 expression in neuron‐differentiated SH‐SY5Y, cultures treated with the indicated drugs for 48 h were fixed with 4% formaldehyde for 15 min at room temperature followed by blocking in 6% BSA‐0.2% Triton‐X100 for 30 min. Primary antibodies (anti‐TDP‐43, Abcam #ab109535; anti‐Tuj1, BioLegend, San Diego, CA, USA, #801202) were incubated at 4 °C overnight and followed by the secondary antibody for 1 h at room temperature. Nuclei were counterstained using Hoechst. Images were taken using InCell 2200 microscope and processed by CellProfiler to measure TDP‐43 signal intensity and nuclei count.

### Western blotting

RIPA buffer with protease inhibitor (Sigma) was used for preparing protein lysates. WB was performed following a standard protocol with the indicated antibodies: TDP‐43 (1 : 1000, Abcam, ab41881), TRIM28 (1 : 1000, Abcam, ab22553), vinculin (1 : 10 000, Abcam, ab129002), PARP1 (1 : 1000, Cell Signaling, Danvers, MA, USA, #9542), β‐actin (1 : 1000, Abcam, ab13822). Signals were visualized by chemiluminescence (ECL, Thermo Scientific, 34,076) and imaged on an Amersham Imager 600 (GE Healthcare, Chicago, IL, USA).

### CRISPR screen

U2OS^T43^ cells were first made to stably express the *S. pyogenes* Cas9 nuclease. In brief, parental cells were lentivirally transduced with pLenti‐Cas9‐T2A‐Blast‐BFP to express a codon‐optimized, WT SpCas9 flanked by two nuclear localization signals linked to a blasticidin‐S‐deaminase—mTagBFP fusion protein via a self‐cleaving peptide (derived from lenti‐dCAS9‐VP64_Blast, a gift from Feng Zhang, Addgene #61425). Following blasticidin selection, a BFP‐high population was sorted twice, and cells were immediately expanded and transduced with the genome‐wide Brunello sgRNA library [22] . The CRISPR guide library was resynthesized to include Unique Molecular Identifiers [23]. Guides were cloned in the pool (oligos synthesized by CustomArray) and packaged into lentivirus (Brunello‐UMI virus). The lentiviral backbone was based on lentiGuide‐Puro (Addgene # 52963), with AU‐flip as described in [24]. Functional titer of the Brunello‐UMI virus in U2OS^T43^ cells was determined by serial dilution of the virus in 6‐well plates followed by puromycin selection. Cas9‐expressing U2OS^T43^ cells were then transduced with Brunello‐UMI virus, in two replicates, at an approximate MOI of 0.4 and 1000 cells per guide in 2 μg·mL^−1^ polybrene. Transduced cells were selected with puromycin (2 μg·mL^−1^) from post‐transduction days two to seven and then kept in culture with or without doxycycline (10 ng·mL^−1^) for 10 days. Cell number per replicate never dropped below 63 × 10^6^, and cells were grown in DMEM +10% tet‐free FBS. Genomic DNA was isolated using the QIAamp DNA Blood Maxi kit (Qiagen 51192), and guide and UMI sequences were amplified by PCR as described in [23]. NGS data were analysed with the mageck software [25] and by UMI lineage dropout analysis [23]. Gene ontology analysis was carried out using the STRING database [26] .

### Quantification and statistical analysis

Data were collected in Excel (Microsoft) and analysed in GraphPad Prism 9 unless specified otherwise. Statistical details of each experiment can be found in the figure legends. All results are representative of at least three independent experiments unless otherwise specified and are presented as mean ± standard error of the mean (SEM). Differences between two groups were analysed using a Student's *t*‐test. Multiple group comparisons were performed using a one‐way or two‐way analysis of variance (ANOVA) followed by *post hoc* tests. Significance is indicated by asterisks: *P < 0.05; **P < 0.01; ***P < 0.001 and ****P < 0.0001.

## Results

### A chemical screen to identify modulators of TDP‐43 toxicity

**Fig. 1:**
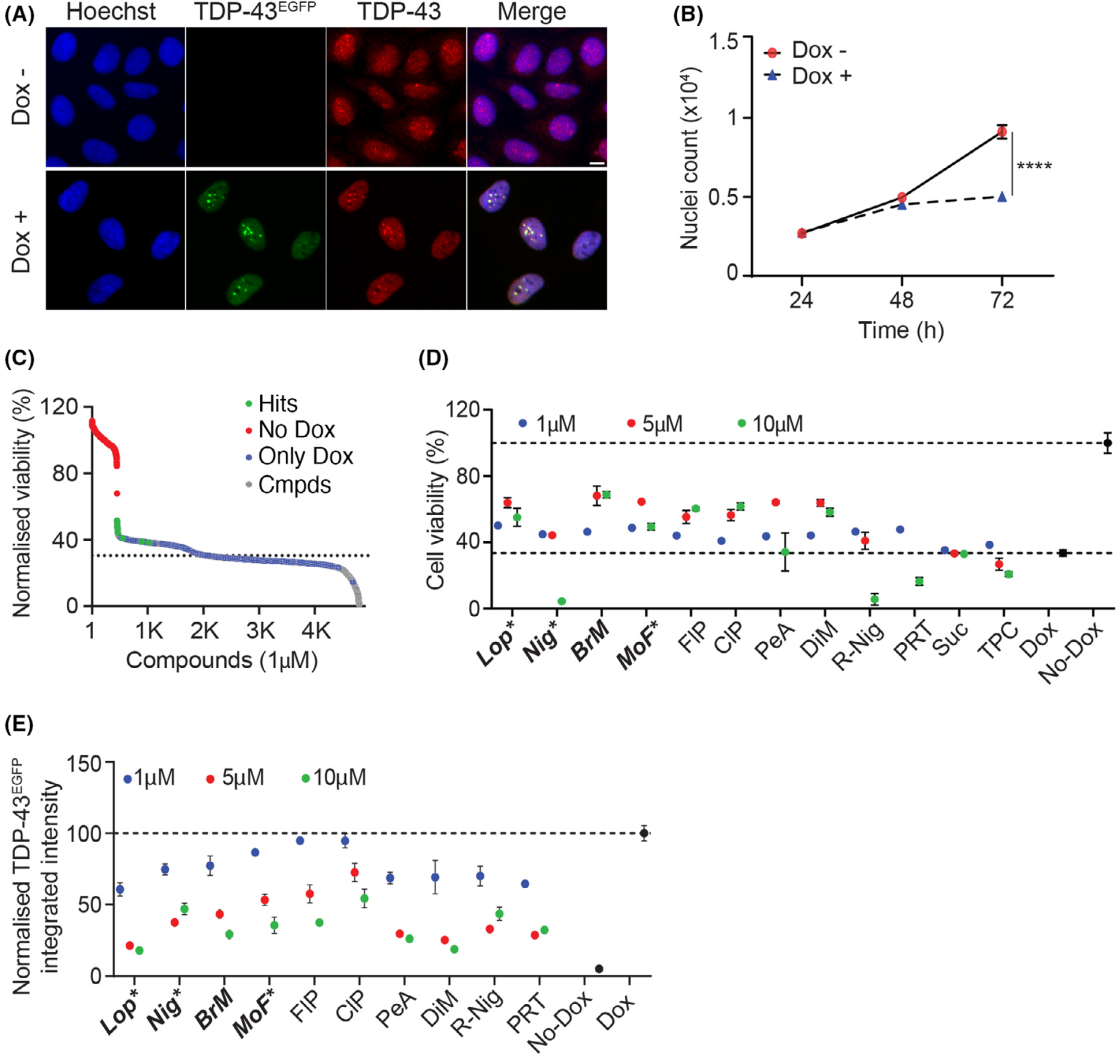
A chemical screen for modulators of TDP‐43 toxicity. (A) Representative images of U2OS^T43^ cells stained with antibodies against TDP‐43 (red) after treatment with Dox (10 ng·mL^−1^) for 72 h. TDP‐43^EGFP^ expression was quantified by the EGFP signal. Hoechst was used to stain nuclei (blue). Scale bar, 5 μm. (B) High‐throughput microscopy (HTM)‐mediated quantification of nuclei counts in U2OS^T43^ cells grown in the presence (blue) or absence (red) of Dox (10 ng·mL^−1^) for the indicated times. Data represent the mean ± SEM (*n* = 3 biological replicates). ****P < 0.0001 by the unpaired *t*‐test. (C) Distribution of the hits from the screen described in Fig. S1E. Compounds with a *Z*‐score > 2 were considered as hits. Data from cells treated only with Dox (red; only Dox) or with no Dox (blue; no Dox) are highlighted. The dashed line represents the mean of negative controls within the different plates. (D) Viability of U2OS^T43^ cells, measured by HTM‐mediated nuclei count 72 h after being treated with Dox (10 ng·mL^−1^) and the indicated compounds at three independent concentrations (1, 5 and 10 μm) for the last 48 h. Data are normalized to untreated cells and represent the mean ± SEM (*n* = 6 biological replicates). A list of the abbreviated compounds is reported in Table S2. (E) TDP‐43^EGFP^ levels in U2OS^T43^ cells quantified by HTM using EGFP signal, 72 h after being treated with Dox (10 ng·mL^−1^) and the indicated compounds at three independent concentrations (1, 5 and 10 μm) for the last 48 h. Data are normalized to untreated cells and represent the mean ± SEM (*n* = 6 biological replicates).

Taking advantage of the T‐REx tetR‐regulated system, we established a human osteosarcoma U2OS cell line (U2OS^T43^) that allowed for the inducible expression of a fusion between TDP‐43 and EGFP (TDP‐43^EGFP^). First, we defined the minimal concentration of doxycycline (Dox), which could be used to induce TDP‐43^EGFP^ expression without any detectable effects on cell growth or morphology in control cells (10 ng·mL^−1^, 72 h). The use of higher Dox concentrations did not substantially increase TDP‐43^EGFP^ expression or cellular toxicity in U2OS^T43^ cells, suggesting that the system was already saturated at 10 ng·mL^−1^ (Fig. S1A,B). Exposure of U2OS^T43^ cells to Dox led to a clear increase in TDP‐43 levels, which accumulated preferentially in nuclear aggregates, as reported in other *in vitro* models, as well as in patient's postmortem specimens [2, 27, 28, 29] (Fig. 1A). Consistent with previous results [30], TDP‐43^EGFP^ overexpression limits cell growth by triggering apoptosis, as evidenced by PARP cleavage (Fig. 1B, Fig. S1C). By contrast, cell viability was not affected in U2OS cells upon the inducible expression of EGFP (U2OS^EGFP^), indicating that apoptosis is specifically driven by TDP‐43 overexpression (Fig. S1D).

Using U2OS^T43^ cells, we next conducted a High‐Throughput Microscopy (HTM)‐mediated chemical screen where we evaluated the activity of 4221 pharmacologically active compounds (including 2969 medically approved) in modulating the toxicity associated with TDP‐43^EGFP^ overexpression. To this end, U2OS^T43^ were seeded in 384‐well plates together with 10 ng·mL^−1^ of Dox, and the compounds from the library were dispensed at 1 μm on the following day for additional 48 h. As a readout for toxicity/viability, the screening pipeline quantified the number of nuclei stained by Hoechst at the 72 h endpoint (Fig. S1E). This experiment identified 17 hit compounds, defined as those increasing nuclei counts more than 2 standard deviations over control wells treated only with Dox (Fig. 1C and Table S1). From the 17 hits, only 12 were unique as the others were redundant in the chemical library. Subsequent validation experiments confirmed that 10 out of 12 compounds rescued TDP‐43 toxicity in a dose‐dependent manner (Fig. 1D). However, a similar dose‐dependent effect of these compounds was also observed in reducing the expression of TDP‐43^EGFP^ in response to Dox (Fig. 1E **)**, suggesting that the observed effects could be due to a selective interference of the drugs with the T‐REx system.

### Effects of medically approved drugs on the tetR system

The previous results indicated that the hit compounds from our screening could be antagonists of the Dox in the tetR system and thus selectively inhibiting the expression of TDP‐43^EGFP^. Consistently, none of the 10 hit compounds reduced the levels of endogenous TDP‐43 levels in the absence of Dox (Fig S2A **).** To evaluate the impact of this phenomenon, from this point we focussed our study on the 4 compounds that had a bigger impact on decreasing TDP‐43^EGFP^ expression: loperamide (Lop), niguldipine (Nig), bromocriptine mesylate (BrM) and mometasone furoate (MoF). Of note, cells expressing TDP‐43^EGFP^ presented lower constitutive levels of endogenous TDP‐43, a fact that has been noted previously [31, 32, 33]. This effect is due to an autoregulatory negative feedback loop related to the binding of TDP‐43 to the 3′UTR of its own mRNA [34]. Of note, while the levels of endogenous TDP‐43 are not affected by the hit compounds in the absence of Dox, we observed a small reduction in endogenous TDP‐43 when Lop, Nig and BrM were used together with Dox (Fig. S2B–D). In any case, given the absence of an effect of these compounds in endogenous TDP‐43 expression in the absence of Dox, we believe that this observation might reflect an indirect effect of the drugs in destabilizing TDP‐43^EGFP^/TDP‐43 complexes. Importantly, endogenous TDP‐43 levels were also not significantly affected by Lop, BrM or Nig in human SH‐SY5Y cells differentiated into neurons (Fig. S2E,F).

**Fig. 2:**
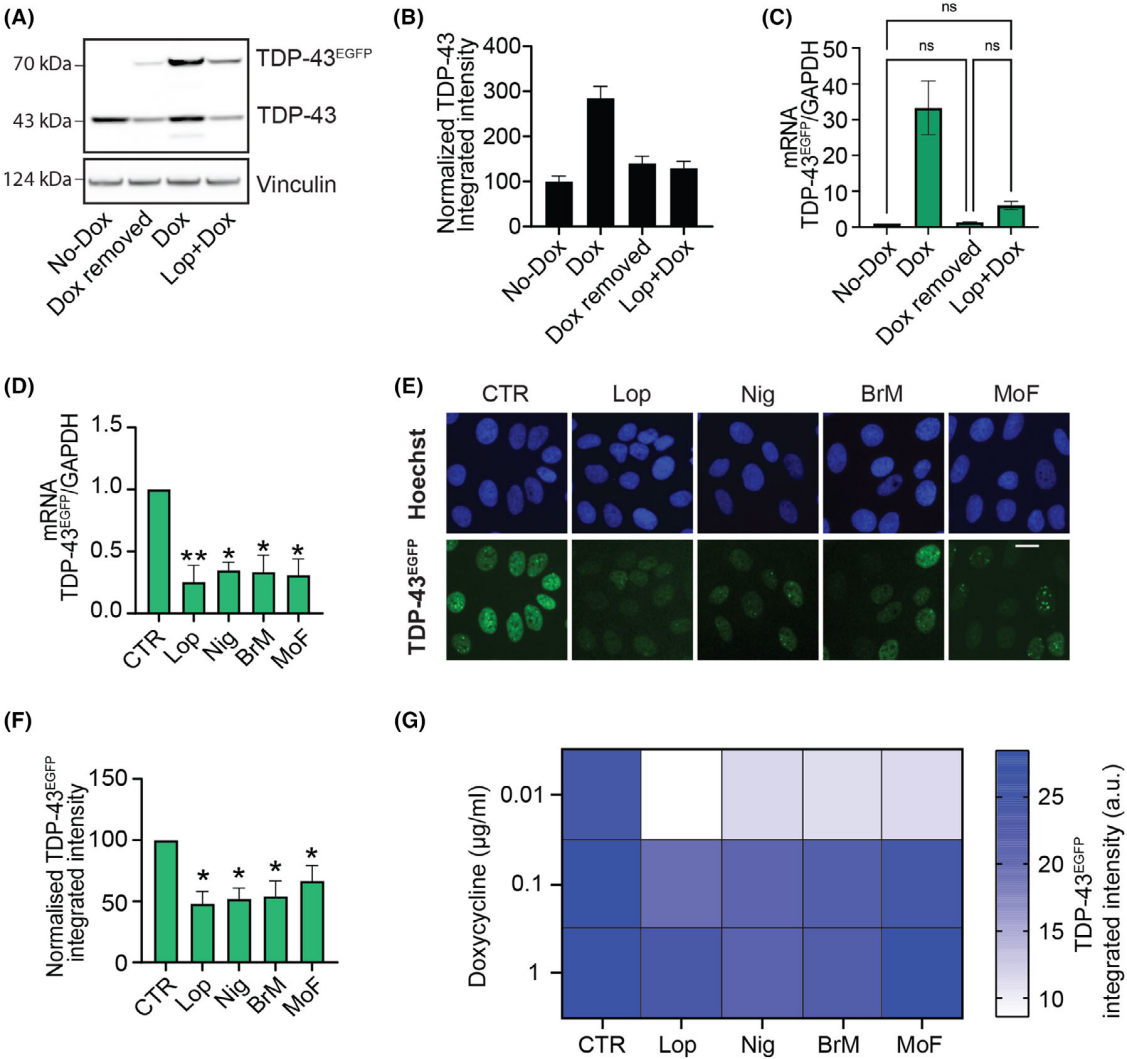
Hit compounds antagonize dox‐dependent induction of TDP‐43^EGFP^. (A) WB illustrating the levels of TDP‐43 and TDP‐43^EGFP^ in U2OS^T43^ cells 72 h after being grown in the absence (no Dox, line 1) or presence of Dox (line 3). In line 2, Dox was removed from the media after 24 h (Dox removed) and cultured for further 48 h, while in line four cells were treated for 24 h with Dox and further 48 h with Lop (Lop + Dox). Vinculin levels are shown as a loading control. Data are representative of two independent experiments. (B) TDP‐43 levels evaluated by HTM with an antibody against TDP‐43 in U2OS^T43^ cells treated as in (A). Data represent the mean ± SEM (*n* = 2 biological replicates) of TDP‐43 integrated intensity normalized on the untreated control (no Dox). (C) TDP‐43^EGFP^ mRNA levels quantified by RT–qPCR using specific primers for EGFP detection, in U2OS^T43^ cells treated as in (A). GAPDH levels were used to normalize expression levels to that of a housekeeping gene. Data represent the mean ± SEM (*n* = 2 biological replicates) of EGFP expression normalized on the untreated control (no Dox). Statistical analysis has been performed by one‐way ANOVA with the Tukey's multiple comparison test. (D) TDP‐43^EGFP^ mRNA levels quantified by RT–qPCR in U2OS^T43^ cells after 4 h of Dox alone (CTR) or in combination with the indicated compounds (Lop, Nig, BrM and MoF). GAPDH levels were used to normalize expression levels to that of a housekeeping gene. Data are presented as mean ± SEM from at least two independent experiments. **P < 0.005, *P < 0.05 by two‐way ANOVA with the Dunnett's multiple comparisons test. (E) Representative images of TDP‐43^EGFP^ (green) in U2OS^T43^ cells treated with Dox alone or with the indicated for 8 h. Hoechst was used to stain nuclei (blue). Scale bar, 10 μm. (F) HTM‐mediated quantification of TDP‐43^EGFP^ levels from an experiment performed as in (E). Data represent the mean ± SEM (*n* = 3 biological replicates). *P < 0.05 by one‐way ANOVA with the Dunnett's multiple comparisons test. (G) Heatmap of TDP‐43^EGFP^ expression levels quantified by HTM in U2OS^T43^ cells exposed to increasing concentrations of Dox with or without the indicated compounds. Data represent the mean ± SEM (*n* = 3 biological replicates). *P* < 0.005 (CTR vs Lop), *P* < 0.05 (CTR vs Nig, BrM, MoF) by two‐way ANOVA with the Bonferroni's multiple comparisons test.

As further support of the antagonistic properties of these compounds, the effect of adding Lop in reducing TDP‐43^EGFP^ expression was equivalent to that of removing Dox, at both protein and mRNA levels (Fig. 2A–C; Fig. S3A). Next, given that the half‐life of Dox is 24 h, we evaluated whether the selected compounds were able to counteract the effect of Dox on TDP‐43 transcription at shorter times. Although TDP‐43 nuclear aggregation and cell death require longer Dox exposures, Dox‐dependent TDP‐43^EGFP^ transcription was already detected after 4 h of exposure, and this was significantly abrogated by the 4 compounds (Fig. 2D). Consistently, TDP‐43^EGFP^ protein levels were significantly reduced by all compounds after 8 h of treatment (Fig. 2E,F). The antagonistic effect of the compounds was reverted by increasing the dose of Dox, indicating the mechanism by which these drugs interfere with the Dox system is competitive (Fig. 2G).

**Fig. 3:**
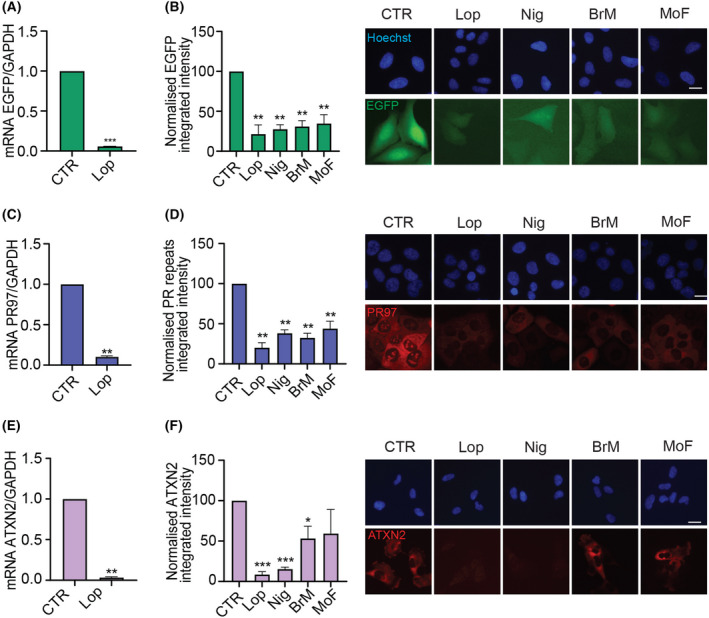
Antagonistic effect of the hit compounds in three independent Tet‐on models. (A) EGFP mRNA expression in U2OS^EGFP^ cells after 4 h of Dox (10 ng·mL^−1^) alone (CTR) or together with Lop. GAPDH was used as housekeeping gene expression control. Data are presented as mean ± SEM from two independent experiments. ***P < 0.001 by the one‐sample t‐test. (B) HTM‐mediated evaluation of EGFP levels in U2OS^EGFP^ cells, monitored by quantification of EGFP signal, 8 h after exposure to Dox alone (CTR) or together with the indicated compounds. Data represent the mean ± SEM (*n* = 3 biological replicates) of the total TDP‐43^EGFP^ integrated intensity normalized to the levels found on controls. **P < 0.01 by the one‐sample *t*‐test. Representative images of this analysis are shown on the right. Hoechst was used to stain nuclei. Scale bar, 10 μm. (C) PR97 mRNA expression in U2OS^PR97^ cells after 4 h of Dox (20 ng·mL^−1^) alone (CTR) or together with Lop. GAPDH was used as housekeeping gene expression control. Data are presented as mean ± SEM from two independent experiments. **P < 0.01 by the one‐sample *t*‐test. (D) HTM‐mediated quantification of PR97 levels in U2OS^PR97^ cells, monitored with an antibody against poly(PR) peptides, 8 h after exposure to Dox alone (CTR) or together with the indicated compounds. Data represent the mean ± SEM (*n* = 3 biological replicates) of the total PR97 integrated intensity normalized to the levels found on controls. **P < 0.01 by the one‐sample *t*‐test. Representative images of this analysis are shown on the right. Hoechst was used to stain nuclei. Scale bar, 10 μm. (E) ATXN2 mRNA expression in RPE‐1^ATXN2^ cells after 4 h of Dox (100 ng·mL^−1^) alone (CTR) or together with Lop. GAPDH was used as housekeeping gene expression control. Data are presented as mean ± SEM from two independent experiments. **P < 0.01 by the one‐sample *t*‐test. (F) HTM‐mediated quantification of ATXN2 levels in RPE‐1^ATXN2^ cells, monitored with an antibody against ATXN2, 8 h after exposure to Dox alone (CTR) or together with the indicated compounds. Data represent the mean ± SEM (*n* = 3 biological replicates) of the total ATXN2 integrated intensity normalized to the levels found on controls. ***P < 0.001, *P < 0.05 by the one‐sample *t*‐test. Representative images of this analysis are shown on the right. Hoechst was used to stain nuclei. Scale bar, 10 μm.

To exclude that the antagonistic effects of the compounds were not restricted to a specific gene or dependent on the transgene integration site, we evaluated their impact in 3 additional cell lines harbouring Dox‐inducible expression of different GOIs: (a) U2OS cells with inducible expression of EGFP (U2OS^EGFP^), (b) U2OS with inducible expression of 97 repeats of a PR dipeptide (U2OS^PR97^) and (c) RPE‐1 cells with inducible expression of ATXN2 (RPE‐1^ATXN2^). The 4 compounds significantly reduced the expression of the GOI in the 3 independent systems, with Lop consistently showing the biggest effect (Fig. 3). In addition to the effects not being restricted to the GOI, we also found that the 4 compounds also abrogated the effect of tetracycline (Tet) in inducing TDP‐43^EGFP^ expression in U2OS^T43^ cells (Fig. S3B). Once again, the effect of the compounds in counteracting gene expression was observed in independent TetR cell models and counteracted by increasing the dose of Tet or Dox, further supporting a competitive mechanism of action (Fig. S3C–F). Altogether, these data identify 4 compounds, 3 of which are medically approved, that act as competitive antagonists of Tet and Dox in inducing gene expression in the TetR system.

### A genome‐wide CRISPR screen identifies genetic regulators of TetR system

**Fig. 4:**
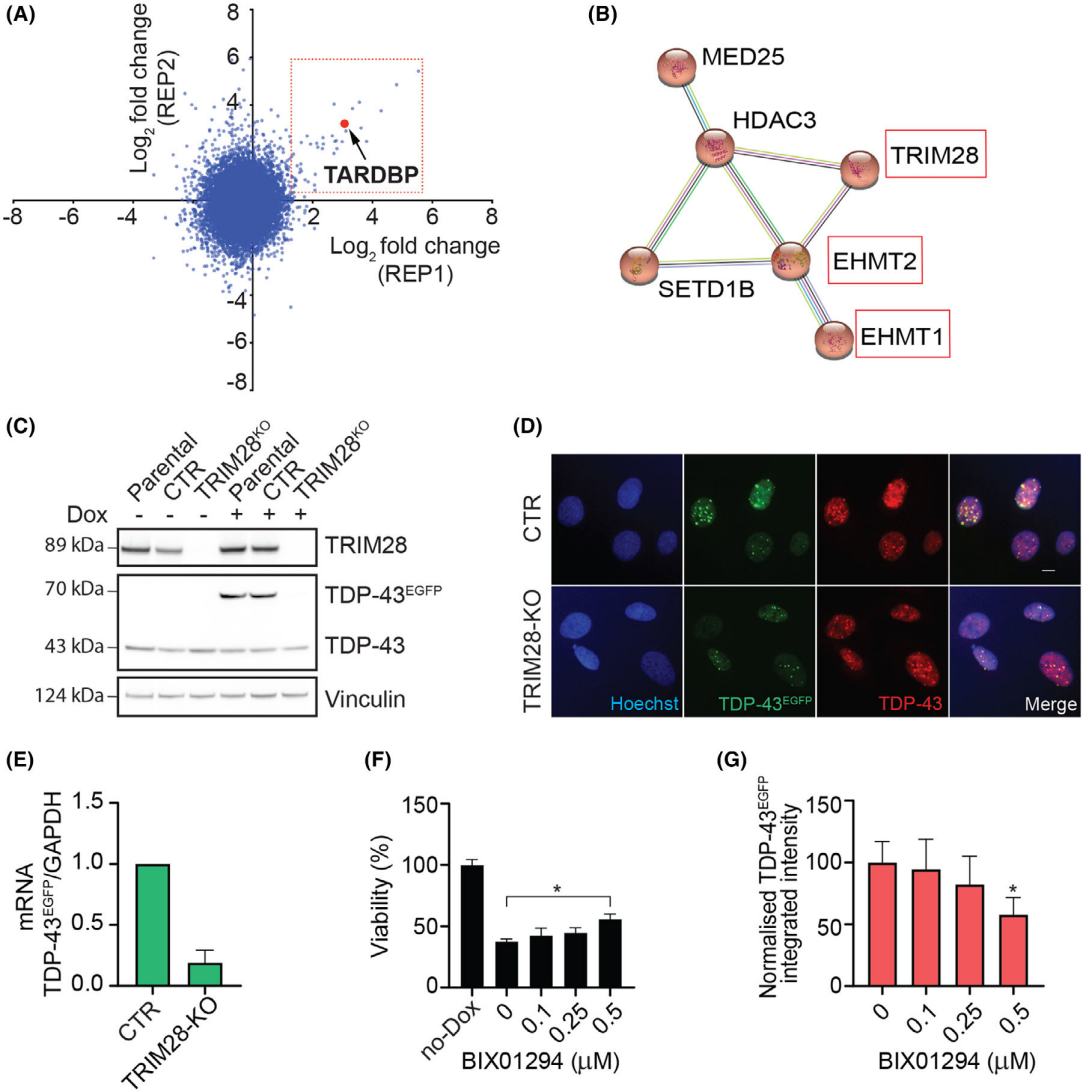
A genome‐wide CRISPR screen identifies epigenetic regulators of the Tet‐on system. (A) The graph represents the abundance of each sgRNA in 2 replicate experiments measured as log2 fold change (lfc) in U2OS^T43^ cells that had been previously infected with the Brunello sgRNA library and grown for 10 days in the presence of Dox. The red dot identifies the sgRNA targeting *TARDBP* (TDP‐43), which was enriched in both experiments. (B) A STRING interaction network illustrating a cluster of chromatin remodelling proteins, which were found to be among the hits in the experiment defined in (A). (C) WB illustrating the absence of TRIM28 expression in TRIM28‐deficient U2OS^T43^ cells generated by CRISPR, 24 h after exposure to Dox. A clone where TRIM28 was not deleted is shown as a control (CTR), together with the parental cell line. Vinculin levels are shown as a loading control. Data are representative of three independent experiments. (D) Representative images of TDP‐43^EGFP^ (green) and TDP‐43 levels (red) in control (CTR) and TRIM28‐deficient (TRIM28‐KO) U2OS^T43^ cells treated with Dox for 24 h. Hoechst was used to stain nuclei (blue). Scale bar, 10 μm. (E) TDP‐43^EGFP^ mRNA levels quantified by RT–qPCR in Control (CTR) and TRIM28‐deficient (TRIM28‐KO) U2OS^T43^ cells after a 4 h of exposure to Dox. GAPDH levels were used to normalize expression levels to that of a housekeeping gene. Data are presented as mean ± SEM from two independent experiments. (F) Viability (measured as the percentage of the number of nuclei observed in each condition, compared to that of cells untreated, no DOX) in U2OS^T43^ cells after 48 h of treatment with BIX01294 at the indicated concentrations, followed by 72 h of Dox. Nuclei counts were quantified by HTM. Data represent the mean ± SEM (*n* = 3 biological replicates). *P < 0.05 by the one‐way ANOVA with multiple comparisons test. (G) HTM‐mediated quantification of TDP‐43^EGFP^ levels in U2OS^T43^ cells after 48 h of treatment with BIX01294 at the indicated concentrations, followed by 72 h of Dox. Data represent the mean ± SEM (*n* = 3 biological replicates) of TDP‐43^EGFP^ integrated intensity normalized on the untreated control. *P < 0.05 by the one‐way ANOVA with multiple comparisons test.

In addition to the chemical screen, we also used U2OS^T43^ cells to perform a forward genome‐wide CRISPR screening to identify genes, deficiency of which is able to mitigate the toxicity associated with TDP‐43 overexpression. To this end, U2OS^T43^ cells were stably transfected with the *S. pyogenes* Cas9 nuclease and subsequently transduced at a multiplicity of infection (MOI) of ~ 0.5 with lentiviruses carrying the Brunello sgRNA library, which comprises 77 441 sgRNAs (an average of 4 per gene) and 1000 nontargeting control sgRNAs [22]. After selecting infected cells, these were grown with or without doxycycline for 10 days. At this point, sgRNAs were amplified from genomic DNA and sequenced in order to identify those significantly enriched in TDP‐43 overexpressing cells (Fig. S4). While we are still validating the results of the screen, one of the top hits that conferred resistance was TDP43 (*TARDBP*) itself, supporting the usefulness of the approach (Fig. 4A). In any case, based on gene ontology analyses we identified a hit node with genes that are involved in epigenetic regulation, which included TRIM28, EHMT1, EHMT2, SETD1B, HDAC3 and MED25 (Fig. 4B). Triggered by our previous findings in the chemical screen, we nevertheless wondered whether these epigenetic regulators were also affecting Dox‐inducible expression of TDP‐43^EGFP^ in U2OS^T43^ cells.

Among this set, sgRNAs targeting TRIM28 were ranked as the highest enriched on the screen. Thus, we first generated a TRIM28‐deficient U2OS^T43^ cell line (Fig. 4C). WB and High‐Throughput Microscopy analyses revealed that Dox‐inducible TDP‐43^EGFP^ expression was significantly reduced in TRIM28‐deficient U2OS^T43^ cells compared with the parental cell line, as revealed by both western blot and immunofluorescence (Fig. 4C,D). The reduction in Dox‐induced TDP‐43^EGFP^ expression was also observed at the transcriptional level (Fig. 4E). Next, we tested the potential relevance of EHMT1 and EHMT2, part of the G9a methyltransferase complex that catalyzes histone H3 mono‐ and dimethylation at lysine 9 and 27, as sgRNAs targeting both genes were identified as hits in our screen. To this end, we used the small‐molecule inhibitor BIX01294, which targets the G9a complex. While the use of BIX01294 significantly limited the toxicity of TDP‐43^EGFP^ overexpression in U2OS^T43^ cells (Fig. 4F), later experiments revealed that this was again due to a reduction in the expression of the transgene (Fig. 4G **)**. Together, these experiments identified perturbations that limit the efficiency of the Tet‐On system and highlight the limitations of this approach in conducting genetic or chemical screens in mammalian cells.

## Discussion

Even though ALS is driven by many independent mutations, a common hallmark is the accumulation of TDP‐43 aggregates [35]. Accordingly, and similar to what has been a driving force in the search of a cure for other neurological disorders such as Alzheimer's disease, many efforts have been placed in trying to identify chemicals capable of reducing the aggregates. In this regard, while several chemical and genetic screens have been conducted to identify modulators of TDP‐43 distribution [28, 31, 36], ours is the first to use TDP‐43‐driven toxicity as a readout of the assay. To this end, we chose to induce TDP‐43^EGFP^ expression using a tet‐regulated expression system, which is arguably the most widely used approach to induce or repress the expression of a GOI in mammalian cells [11]. Unfortunately, after screening more than 4000 compounds, including the vast majority of medically approved drugs, none showed a significant effect in mitigating the toxicity driven by TDP‐43 overexpression, raising doubts as to whether drug repositioning efforts might succeed in this regard. Nevertheless, we are aware of the limitations of our approach and it is certainly possible that other screenings of TDP‐43 toxicity based on different model systems (e.g. patient‐derived motor neurons) could still find valid drugs in this collection.

All primary hits identified in our screen were later found to be antagonists of tetracycline antibiotics. This information is nevertheless relevant as (a) it raises awareness on the limitations of using the Tet‐On/Tet‐Off system to conduct chemical screens and (b) some of these compounds are medically approved and our findings suggest that their use could modulate the efficacy of antibiotics when jointly administered. In fact, Lop was independently identified in another screen oriented to discover modulators of antibiotic efficacy through combination with nonantibiotic drugs [37]. Besides chemicals, our study has also identified TRIM28 and the G9a histone methyltransferase regulate transcriptional induction in the Tet‐On system, highlighting the relevance of considering the epigenetic regulation of this system. In summary, we here provide a resource that illustrates the limited impact of medically approved drugs in modulating the toxicity associated with TDP‐43 overexpression and reveal new chemical and genetic regulators of the Tet‐On system in mammalian cells.

## Conflict of interest

The authors declare no conflict of interest.

## Author contributions

OF, DH and VC involved in conceptualization; OS, BP, MB, BP and VC involved in methodology; VC, MH, XL and LL involved in investigation; OF and VC involved in writing; OF, DH and JC involved in supervision.

## Supporting information


**Fig. S1.** TDP‐43 overexpression reduces cell viability by triggering apoptosis. (A) Viability of U2OS^T43^ cells measured by quantifying nuclei through HTM after 72 hrs of exposure to the indicated Dox concentrations. Data are normalized to untreated cells and represent the mean ± SEM (n = 6 biological replicates). ****p<0.0001 by one‐way ANOVA with Dunnett's multiple comparisons test. (B) TDP‐43^EGFP^ levels in U2OS^T43^ cells quantified by HTM using EGFP signal, 72 hrs after being treated with Dox at the indicated concentrations. Data represents the mean ± SEM of the EGFP integrated intensity measured in 5 independent experiments. (C) WB illustrating the levels of TDP‐43 and TDP‐43^EGFP^ in U2OS^T43^ cells after 72 hrs of Dox associated with PARP cleavage as a readout of apoptosis. b‐actin levels are shown as a loading control. (D) Viability of U2OS^EGFP^ cells measured by quantifying nuclei through HTM after 72 hrs of exposure to the indicated Dox concentration. On the right, WB showing endogenous TDP‐43 and any cleavage of PARP are reported. b‐actin levels are shown as a loading control. (E) Schematic overview of the screening workflow. U2OS^T43^ cells were seeded together with Dox (10 ng/ml) in triplicate plates. After 24 hrs, compounds from the chemical library were dispensed at a final 1μM concentration. 48 hrs after exposure, cells were fixed and stained with Hoechst to enable the quantification of nuclei numbers by HTM.
**Fig. S2.** Effect of the hit compounds on endogenous and Dox‐dependent TDP‐43^EGFP^ levels. (A) TDP‐43 levels in U2OS^T43^ cells quantified by HTM using an antibody against endogenous TDP‐43, after 72 hrs after being treated with Dox or after the indicated compounds at three independent concentrations (1, 5 and 10μM) without doxycycline. Data are normalized to Dox treated cells (Dox) and represent the mean ± SEM (n = 6 biological replicates). (B) WB illustrating the levels of TDP‐43 and TDP‐43^EGFP^ in U2OS^T43^ cells after exposure to Dox for 24 hrs followed by 48 hours of Lop at the indicated concentrations. Vinculin levels are shown as a loading control. (C) WB illustrating the levels of TDP‐43 and TDP‐43^EGFP^ in U2OS^T43^ cells after exposure to Dox for 24 hrs followed by 48 hours of Nig at the indicated concentrations. Vinculin levels are shown as a loading control. (D) WB illustrating the levels of TDP‐43 and TDP‐43^EGFP^ in U2OS^T43^ cells after exposure to Dox for 24 hrs followed by 48 hours of BrM at the indicated concentrations. Vinculin levels are shown as a loading control. (E) TDP‐43 levels in neurons differentiated from SH‐SY5Y cells as quantified by HTM using an antibody against endogenous TDP‐43, 48 hrs after being treated with the indicated compounds. (F) Representative image from the HTM analysis defined in E, revealing the neuronal morphology of the SH‐SY5Y cultures stained with antibodies against TDP‐43 (red) and b3‐TUBULIN (TUB, green). DNA was stained with Hoechst (blue). Scale bar (white) indicates 10 μm.
**Fig. S3.** A dose‐dependent antagonistic effect of hit compounds on Dox‐ and tet‐dependent induction of gene expression. (A) TDP‐43 mRNA levels quantified by RT‐qPCR in U2OS^T43^ cells treated as in Fig. 2C. GAPDH levels were used to normalize expression levels to that of a housekeeping gene. Data represents the mean ± SEM (n = 2 biological replicates) of TDP‐43 expression normalized on the untreated control (No‐Dox). Statistical analysis has been performed by one‐way ANOVA with Tukey's multiple comparison test. (B) Heatmap of TDP‐43^EGFP^ expression levels quantified by HTM in U2OS^T43^ cells exposed to increasing concentrations of tet with or without the indicated compounds. Data represents the mean ± SEM (n = 3 biological replicates). (C) Heatmap of EGFP expression levels quantified by HTM in U2OS^EGFP^ cells exposed to increasing concentrations of Dox with or without the indicated compounds. Data represents the mean ± SEM (n = 3 biological replicates). (D) Heatmap of EGFP expression levels quantified by HTM in U2OS^EGFP^ cells exposed to increasing concentrations of tet with or without the indicated compounds. Data represents the mean ± SEM (n = 3 biological replicates). (E) Heatmap of PR97 expression levels quantified by HTM in U2OSPR97 cells exposed to increasing concentrations of Dox with or without the indicated compounds. Data represents the mean ± SEM (n = 3 biological replicates). (F) Heatmap of PR97 expression levels quantified by HTM in U2OSPR97 cells exposed to increasing concentrations of tet with or without the indicated compounds. Data represents the mean ± SEM (n = 3 biological replicates).
**Fig. S4.** Workflow of the CRISPR screen performed in U2OS^T43^ cells. Schematic overview of the forward genome‐wide CRISPR screen conducted in this study. Briefly, U2OS^T43^ cells stably expressing Cas9 were transduced with a pooled sgRNAs library at a low MOI. Transduced cells were then grown in the absence or presence of Dox for 10 days. At this point, the abundance of each sgRNA in the remaining cell populations was calculated by next generation sequencing of PCR‐amplified sgRNA sequences. The experiment was performed in 2 independent biological replicates and enrichment scores calculated independently from each of them.

**Table S1.** List of compounds rescuing the viability of TDP‐43EGFP overexpressing cells.
**Table S2.** Compound abbreviations.
**Table S3.** Source of reagents.

## Data Availability

The data that support the findings of this study are available in the main figures and supplementary material of this article.
