# Supplementary material for: New regulators of the tetracycline‐inducible gene expression system identified by chemical and genetic screens
Source: FEBS Open Bio. 2022 Sep 11;12(10):1896–908. doi: 10.1002/2211-5463.13482 (PMC9527584; doi:10.1002/2211-5463.13482)
Supplement: Supplementary file 2 — Table S1. List of compounds rescuing the viability of TDP‐43EGFP overexpressing cells. Table S2. Compound abbreviations. Table S3. Source of reagents. [file FEB4-12-1896-s001.pdf]

Table S1. List of compounds rescuing the viability of TDP-43^{EGFP} overexpressing cells.

Compound ID	Batch ID	Library	Compound Name	Therapeutic class	Normalised nuclei count	Standard deviation	Normalised nuclei count Neg CTR	Standard deviation	Normalised nuclei count Pos CTR	Standard deviation	Z-score
CBK041816C	BJ1834168	Enzo	Loperamide·HCl	Ca2+ Blocker	47,12791039	2,812351	29,12312822	2,0274601	100	4,5146193	3,015705
CBK041816C	BJ1834412	Enzo	Loperamide·HCl	Calcium channel	44,26219218	2,616922	27,76060287	2,0364995	100	3,9336966	3,436632
CBK290434	BJ1834409	Enzo	Niguldipine	Calcium channel	40,93674741	3,180713	27,76060287	2,0364995	100	3,9336966	2,75156
CBK041816C	BA1064144	Prestwick	Loperamide hydrochloride	Gastroenterology	38,92630139	2,122018	26,93567388	0,9074579	100	6,8693939	4,310168
CBK041816C	BJ1836074	Tocris mini	Loperamide hydrochloride	Peripherally acting µ agonist. Also Ca2+ channel blocker	36,33694847	4,237543	25,17787127	1,2983054	100	4,3807581	3,312059
CBK290997C	BJ1856299	Selleck-known kinase inhibitors	PRT062607 (P505-15, PRT2607, BIIB057) HCl	Syk	50,94246682	6,531776	36,20660095	1,7398738	100	3,1376512	2,399776
CBK289895	BJ1834423	Enzo	Penitrem A	Potassium channel	38,3510138	4,405175	27,76060287	2,0364995	100	3,9336966	2,168616
CBK289950G	BJ1835073	Tocris mini	Dihydroergocristine mesylate	Partial α agonist. Non-selective	51,14583269	2,188903	37,22406109	1,7101686	100	4,5725361	3,49016
CBK200761	BJ1838560	Selleck tool compounds	Mometasone furoate	NA	51,66129052	2,753683	38,86857778	2,0855832	100	3,6186737	2,446069
CBK200761	BA1064572	Prestwick	Mometasone furoate	Endocrinology	35,8651244	0,482834	27,25249107	1,6723928	100	4,0592056	3,366255
CBK289955	BJ1835139	Tocris mini	TPCA-1	Potent, selective inhibitor of IKK-2	48,27023492	0,894071	37,22406109	1,7101686	100	4,5725361	2,731804
CBK200701G	BJ1835054	Tocris mini	Bromocriptine mesylate	Selective D2-like agonist	47,66512828	2,020484	37,22406109	1,7101686	100	4,5725361	2,559436
CBK041149C	BJ1835374	Tocris mini	(R)-(-)-Niguldipine hydrochloride	α1 antagonist, L-type Ca2+ channel blocker. Less active enantiomer of Niguldipine hydrochloride (Cat. No. 1123)	47,60458671	2,163254	37,22406109	1,7101686	100	4,5725361	2,531613
CBK041149C	BJ1835373	Tocris mini	(S)-(+)-Niguldipine hydrochloride	α1 antagonist, L-type Ca2+ channel blocker	46,48957357	2,075105	37,22406109	1,7101686	100	4,5725361	2,251765
CBK200509	BA1064781	Prestwick	Clobetasol propionate	Metabolism	34,03529109	0,858184	27,25249107	1,6723928	100	4,0592056	2,647472
CBK016912	BA1064702	Prestwick	Succinylsulfathiazole	Infectiology	33,22835406	2,782178	27,25249107	1,6723928	100	4,0592056	2,387994
CBK200868	BJ1835649	Tocris mini	Fluticasone propionate	Selective high affinity glucocorticoid agonist	31,64975049	2,58535	26,37414799	1,2533473	100	5,9417387	2,016654

Table S2. Compound abbreviations.

Compound	Abbreviation
Loperamide HCl	Lop
Niguldipine	Nig
Bromocriptine mesylate	BrM
Mometasone furoate	MoF
Fluticasone proprionate	FlP
Clobetasol propionate	ClP
Penitrem A	PeA
Dihydroergocristine mesylate	DiM
(R)-(-)-Niguldipine HCl	R-Nig
PRT062607 HCl	PRT
Doxycycline -	No-Dox
Doxycycline +	Dox

Table S3. Source of reagents.

REAGENT or RESOURCE	SOURCE	IDENTIFIER
Antibodies		
TDP-43	Abcam	Cat# Ab41881
TRIM28	Abcam	Cat# Ab22553
PARP1	Cell Signalling	Cat# 9542
β-actin	Abcam	Cat# Ab13822
Vinculin	Abcam	Cat# Ab129002
PR repeats	Proteintech	Cat# 23979-1-AP
ATXN2	BD Biosciences	Cat# 611378
Anti-rabbit Alexafluor 647	Invitrogen	Cat# A32733
Anti-mouse Alexafluor 647	Invitrogen	Cat# A32728
Bacterial and virus strains		
Brunello-UMI-virus	CustomArray, Genscript	
pLenti-Cas9-T2A-Blast-BFP	derived from lenti-dCAS9-VP64_Blast, a gift from Feng Zhang	Addgene #61425
Chemicals, peptides, and recombinant proteins		
Doxycycline hyclate	Sigma-Aldrich	Cat# D9891
Tetracycline hydrochloride	Sigma-Aldrich	Cat# T7660
Mometasone furoate	Sigma-Aldrich	Cat# M4074
BIX01294 trihydrochloride hydrate	Sigma-Aldrich	Cat# B9311
Loperamide hydrochloride	Enzo Life Sciences	Cat# ALX-550-253
Niguldipine hydrochloride	Enzo Life Sciences	Cat# BML-CA216
Bromocriptine mesylate	Tocris	Cat# 0427
Zeocin	Thermo Fisher	Cat# R25001
Blasticidin	Thermo Fisher	Cat# R21001
Hoechst 33342	Sigma-Aldrich	Cat# 14533
Critical commercial assays		
PureLink™ RNA mini kit	Invitrogen	Cat# 12183018A
SYBR® Green RNA-to-CT™ 1-Step Kit	Thermo Fisher	Cat# 4389986
Lipofectamine 3000	Thermo Fisher	Cat# L300015
Lipofectamine 2000	Thermo Fisher	Cat# 11668019
Experimental models: Cell lines		
T-Rex U2OS	Kind gift from Steve Jackson Laboratory	N/A
hTERT RPE-1	ATCC	Cat# CRL-4000
Oligonucleotides		
ATXN2 F 5’ - CCCAGCAGCACAACAG - 3’	Sigma-Aldrich	N/A
ATXN2 R 5’ - ATGTGGGGTGGGTTGG - 3’	Sigma-Aldrich	N/A
EGFP F 5’ - CTACCCCGACCACATGAAGC - 3’	Sigma-Aldrich	N/A
EGFP R 5’ - AAGAAGATGGTGCGCTCCTG - 3’	Sigma-Aldrich	N/A
PR97 F 5’ - CTAGGCCAAGACCCCGAAC - 3’	Sigma-Aldrich	N/A
PR97 R 5’ - TGCTACACGGTCTAATGCGA - 3’	Sigma-Aldrich	N/A
GAPDH F 5’ - TGCACCACCAACTGCTTAG - 3’	Sigma-Aldrich	N/A
GAPDH R 5’- GGATGCAGGGATGATGTTC - 3’	Sigma-Aldrich	N/A
crRNA TRIM28 TACCAGTAGAGCGCACAGTA	Horizon	Cat# CM-005046-01
tracrRNA	Horizon	Cat# U-002005-05
sgRNA ATXN2 AATCTATGCAAATATGAGGA	Sigma-Aldrich	Shalem et al., 2014
Recombinant DNA		

pINTO-C-FH	Kind gift from Emilio Lecona	N/A
pEGFP-C1	Kind gift from Tatiana Shelkovnikova	Clontech, 6084-1
pEGFP-wtTDP-43	Kind gift from Tatiana Shelkovnikova	N/A
pINTO-EGFP	This paper	N/A
pINTO-wtTDP-43^{EGFP}	This paper	N/A
pINTO-$(PR)_{97}$	This paper	N/A
pcDNA-HA-ATXN2	Kind gift from Daisuke Ito	N/A
Rosa-BleoR-TetON-Snap	This paper	N/A
Rosa-BleoR- TetON-SnapATXN2wt	This paper	N/A
pSpCas9(BB)-2A-GFP (pX458)	Addgene	Cat# 48138
Software and algorithms		
GraphPad Prism 9	Graphpad Software Inc.	GraphPad
Cell Profiler	Kamentsky *et al.* 2011	Cell Profiler
Image J	Schneider *et al.* 2012	ImageJ
KNIME	Berthold *et al.* 2007	KNIME
MaGeCK	Li *et al.* 2014	N/A
UMI lineage dropout	Schmierer *et al.* 2017	N/A
STRING	Szklarczyk *et al.* 2018	STRING
